# Isolated Sigmoid Colon Perforation in the Setting of Blunt Abdominal Trauma: A Case Series

**DOI:** 10.7759/cureus.31591

**Published:** 2022-11-16

**Authors:** Aviral Srivastava, Hari kesh Yadav, Vivek Katiyar

**Affiliations:** 1 Department of General Surgery, Institute of Medical Sciences, Banaras Hindu University, Varanasi, IND

**Keywords:** colon trauma, colostomy, resection anastomosis, sigmoid colon, blunt trauma

## Abstract

Isolated colon injuries following blunt abdominal trauma have been reported with an incidence of 0.1-0.5 %, with sigmoid colon involvement being a rare entity. The sigmoid colon is reportedly involved only in 34.8% of isolated colonic injuries. The most common cause of colonic injuries is motor vehicle accidents. Contrast-enhanced computed tomography has a role in blunt trauma-induced bowel injury evaluation, with 82% accuracy and 64% sensitivity, but its role in the diagnostic evaluation of colonic injuries is controversial.

Surgical treatment comprises primary closure, resection with or without anastomosis, and/or colostomy formation. Primary anastomosis is often favored, while colostomy creation is generally required if devascularized bowel segments present or infrequently in hemodynamic instability scenarios. Subsequent gross presentation, treatment delays due to diagnostic difficulties, and scarcity of management guidelines contribute to high morbidity and mortality. Additional research is required to accurately define patient presentation and explore the benefits of different surgical treatment options.

Hereby is a case series comprising three adult male patients who presented with delayed diffuse severe abdominal pain and distension following blunt abdominal trauma. Computed tomography evaluation in the latter two had findings suggestive of pneumoperitoneum. Post resuscitation, exploratory laparotomy done in each patient denoted isolated sigmoid colon perforation with and without associated mesenteric hematoma. The decision of primary closure, resection with rectosigmoid anastomosis, and resection with end colostomy creation was taken in respective cases based on intraoperative findings of contamination, vascularity, and hemodynamics. Previously documented reports have mentioned findings of associated intra-abdominal solid organ injuries, which were absent in the present case series.

## Introduction

Isolated colon injuries following blunt abdominal trauma have been reported with an incidence of 0.1-0.5 % [1,2], with remote sigmoid colon involvement being a much rarer entity due to the fixity of intraperitoneal sigmoid colon in the pelvic region [2]. Colon involvement in blunt abdominal trauma cases often occurs due to motor vehicle accidents and is frequently associated with other intrabdominal injuries [1,2]. The sigmoid colon is reportedly involved only in one-third of cases of isolated colonic injuries [3]. Diagnostic difficulties and scarcity of specific management guidelines in blunt sigmoid injuries contribute to high morbidity and mortality. Here is a case series of isolated sigmoid colon perforation without associated other intra-abdominal solid or hollow organ involvement.

## Case presentation

Case 1

A 30-year male with a two-day history of diffuse severe abdominal pain, abdominal distension, and obstipation presented following a motor vehicle accident three days ago. No comorbidities/ prior surgical history was stated. The patient was alert, conscious, and oriented; temperature 96.8 F, pulse 116/min, and blood pressure 114/58 mmHg, Glasgow coma scale (GCS) was 15 (E4V5M6) at presentation. On examination, abdominal tenderness and rigidity were present, but shifting dullness or fluid thrill was absent and bowel movements were sluggish. Routine biochemical parameters (complete blood count, liver and renal function test, and PT/INR {prothrombin time/international normalized ratio) were normal. Chest X-ray (PA {posteroanterior} view) and abdominal X-ray (AP {anteroposterior} erect view) were unremarkable. The patient was hemodynamically deranged and was taken for upfront laparotomy without CECT (contrast-enhanced computed tomography) abdomen evaluation as per hospital protocol.

**Figure 1:**
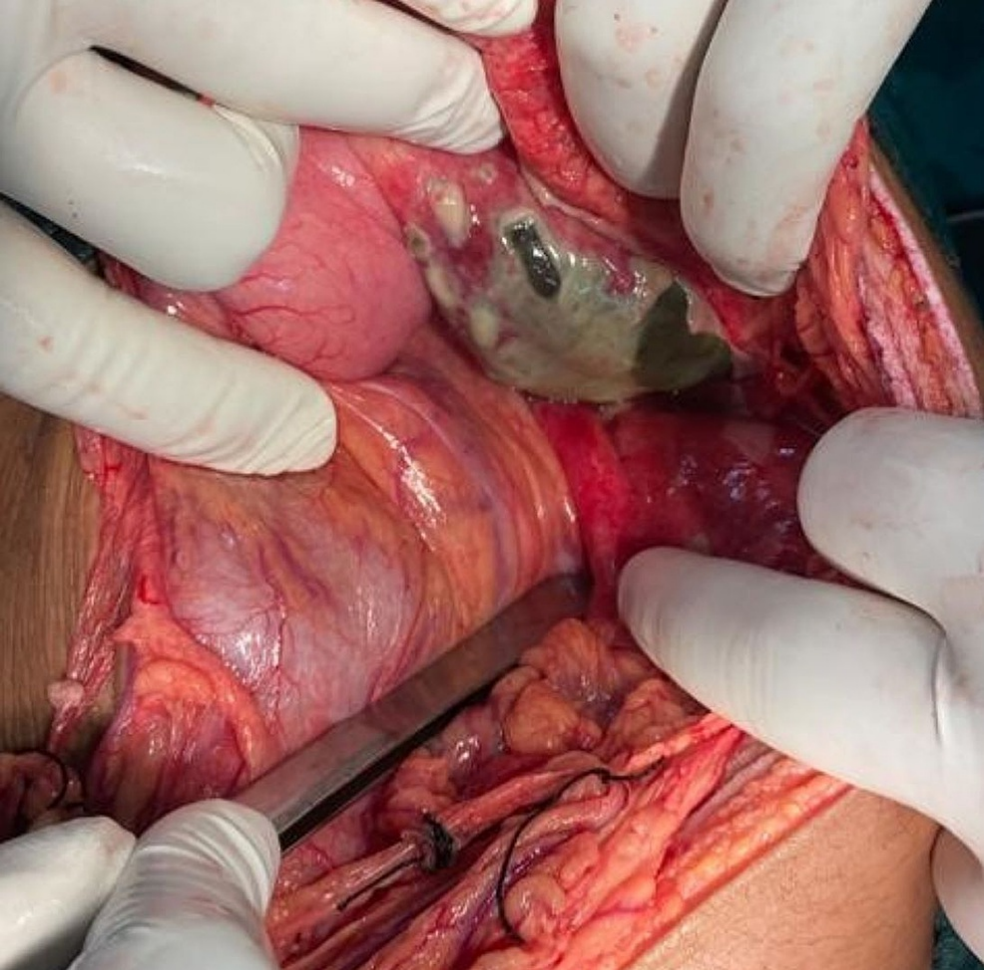
Sigmoid colon perforation The instrument is pointing towards a 1 x 0.5 cm perforation present over the anterior aspect of the distal sigmoid colon at the antimesenteric border, surrounding fecal contamination is seen.

Intraoperatively, 200 ml contaminated intraperitoneal fluid was aspirated and a 1 x 0.5 cm perforation present over the anterior aspect of the distal sigmoid colon at the antimesenteric border was noted (Figure 1). A mesenteric hematoma was present approximately 2 cm proximal to the perforation. Multiple pedunculated polyps in the rectosigmoid region were also noted. Based on the intraoperative assessment, primary repair of perforation (colorrhaphy) was done. The postoperative outcome was unremarkable. Histopathological examination of the polyp revealed juvenile polyposis.

Case 2

A 22-year male presented with a three-day history of lower abdominal pain following a motor vehicle accident six days ago. No known co-morbidities/prior surgical history was given. The patient was alert, conscious, and oriented, temperature 97.8 F, pulse 102/min, blood pressure 118/70 mmHg, and GCS was 15 (E4V5M6). On examination, abdominal tenderness and rigidity were present in the lower quadrant, and bowel movements were normal. Biochemical parameters (complete blood count, liver and renal function test, and PT/INR) were normal. Chest X-ray (PA view) and abdominal X-ray (AP erect view) were unremarkable. 

**Figure 2:**
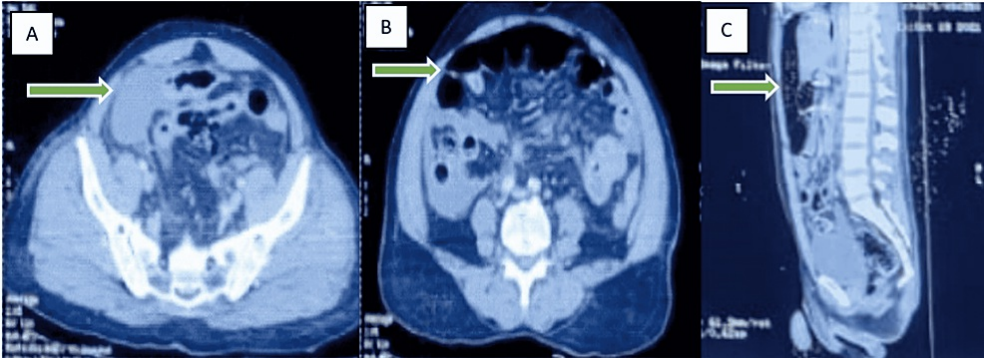
Contrast-enhanced computed tomography suggesting sigmoid colon ischemia Figure 2A: Dilated and fecal-loaded colon loops (arrow) Figure 2B: Dilated proximal bowel loops (arrow) with sigmoid colon edema (suggestive of ischemia) Figure 2C: Dilated bowel loops (arrow) with an air-fluid level

CECT abdomen with intravenous contrast demonstrated an edematous sigmoid colon loop and free fluid in the pelvis (Figure 2), suggestive of sigmoid colonic ischemia. The patient was resuscitated and underwent laparotomy.

**Figure 3:**
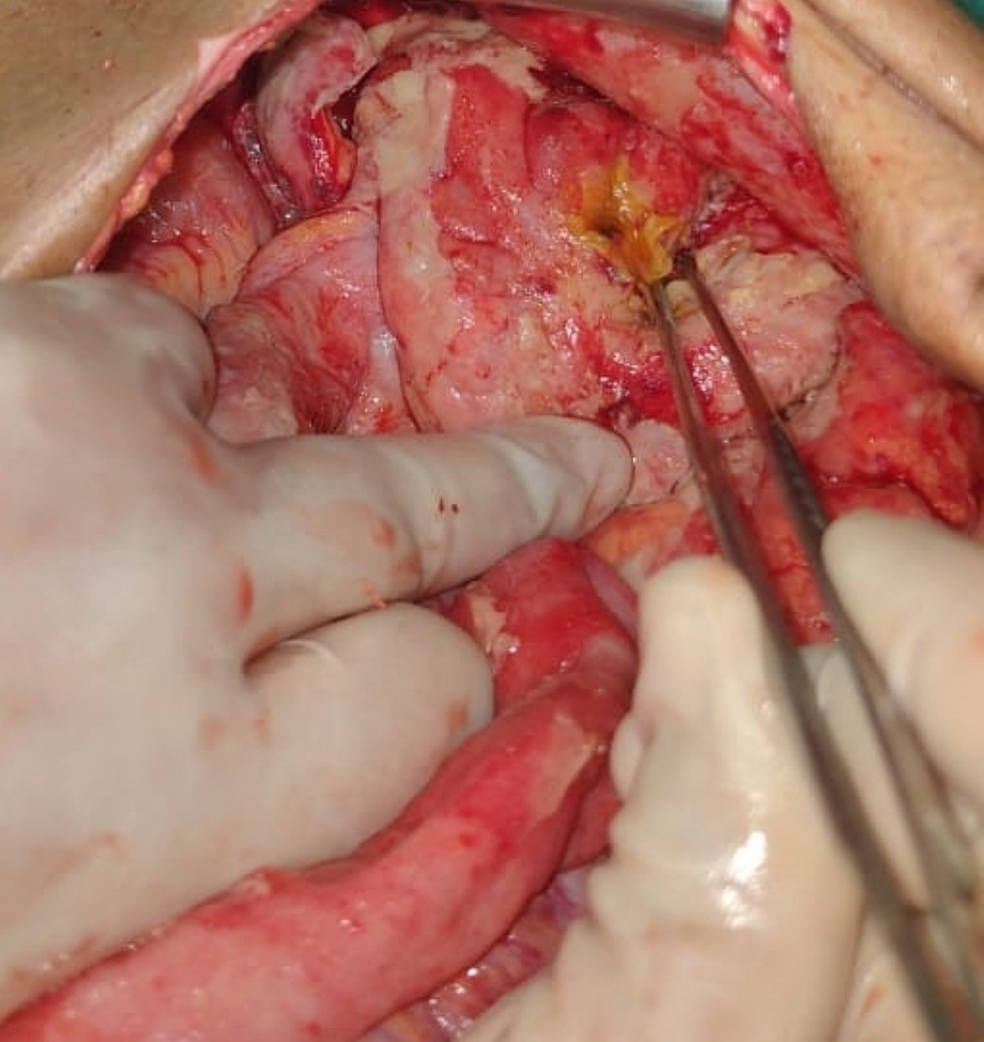
Sigmoid colon perforation 2 x 1 cm perforation over the anterior aspect of the distal sigmoid colon near the antimesenteric border was present, as pointed by the instrument. Gross fecal contamination and membrane formation over surrounding large and small intestine loops were noted.

Intraoperatively, 500 ml sanguineous intraperitoneal fluid was aspirated and gross fecal contamination and membrane formation over large and small intestine areas was noted. A 2 x 1 cm perforation over the anterior aspect of the distal sigmoid colon near the antimesenteric border was present (Figure 3). A 1.5 cm serosal tear was present 5 cm distal to perforation and a mesenteric hematoma was present adjacent to the serosal tear. Owing to adequate hemodynamic stability post-resuscitation and the favorable physiological condition of the patient, the intraoperative decision of resection of the perforated sigmoid colon, followed by rectosigmoid anastomosis with diversion loop ileostomy formation was made. The patient developed superficial SSI (surgical site infection) over a laparotomy wound in post-operative period which was managed by appropriate antibiotics and routine dressing and was discharged after his condition improved. Histopathological examination revealed muscularis propria and subserosal tissue fibrosis and edema.

Case 3

A 64-year male presented with a two-day history of severe lower abdominal pain following a fall from height four days ago. He was on oral medications for Type 2 diabetes mellitus for 5 years with a controlled diabetic profile. He had a history of laparotomy for gastric perforation 18 years ago. The patient was alert, conscious, and oriented, temperature 98.4 F, pulse 118/min, and blood pressure 116/64 mmHg, GCS was 14 (E4V4M6). On examination, abdominal distension, diffuse abdominal tenderness, and rigidity were noted. Shifting dullness was present and bowel movements were sluggish. Leucocytosis (27000 per cubic millimeter) was noted. Chest X-ray (PA view) demonstrated bilateral hemopneumothorax. Abdominal X-ray (AP erect view) was suggestive of possible pneumoperitoneum with distended bowel with multiple air-fluid levels. 

**Figure 4:**
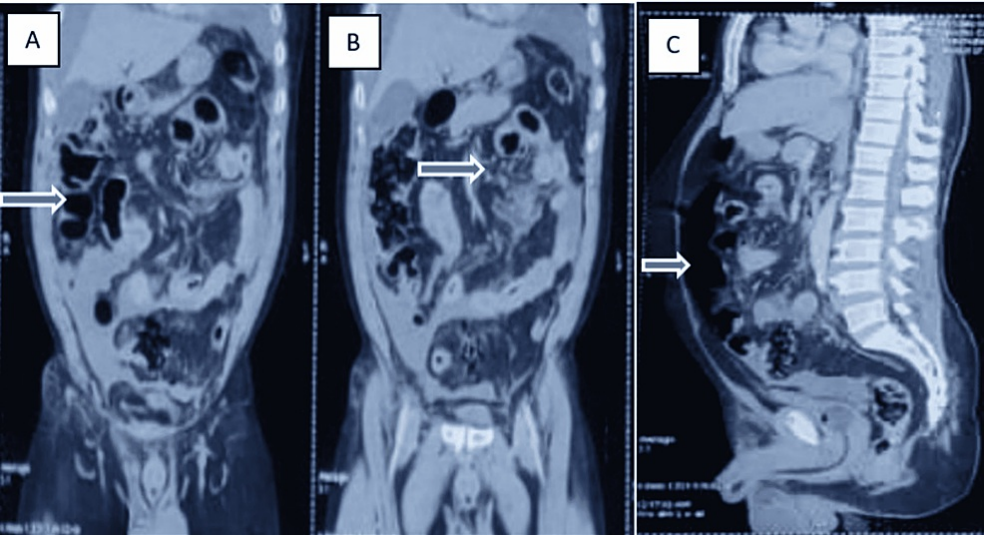
Contrast-enhanced computed tomography demonstrating sigmoid perforation Figure 4A: Hemoperitoneum with mildly distended bowel loops (arrow) Figure 4B: Distended bowel loops (arrow) with contrast extravasation in the sigmoid colon region (sigmoid colon not visualized) Figure 4C: Distended bowel loops and suspected pneumoperitoneum (arrow)

CECT abdomen with intravenous contrast was suggestive of hemoperitoneum and sigmoid colonic ischemia with distended proximal bowel loops, while pneumoperitoneum was suspected and solid organs appeared normal (Figure 4). Bilateral intercostal drainage tubes were placed and the patient underwent laparotomy.

**Figure 5:**
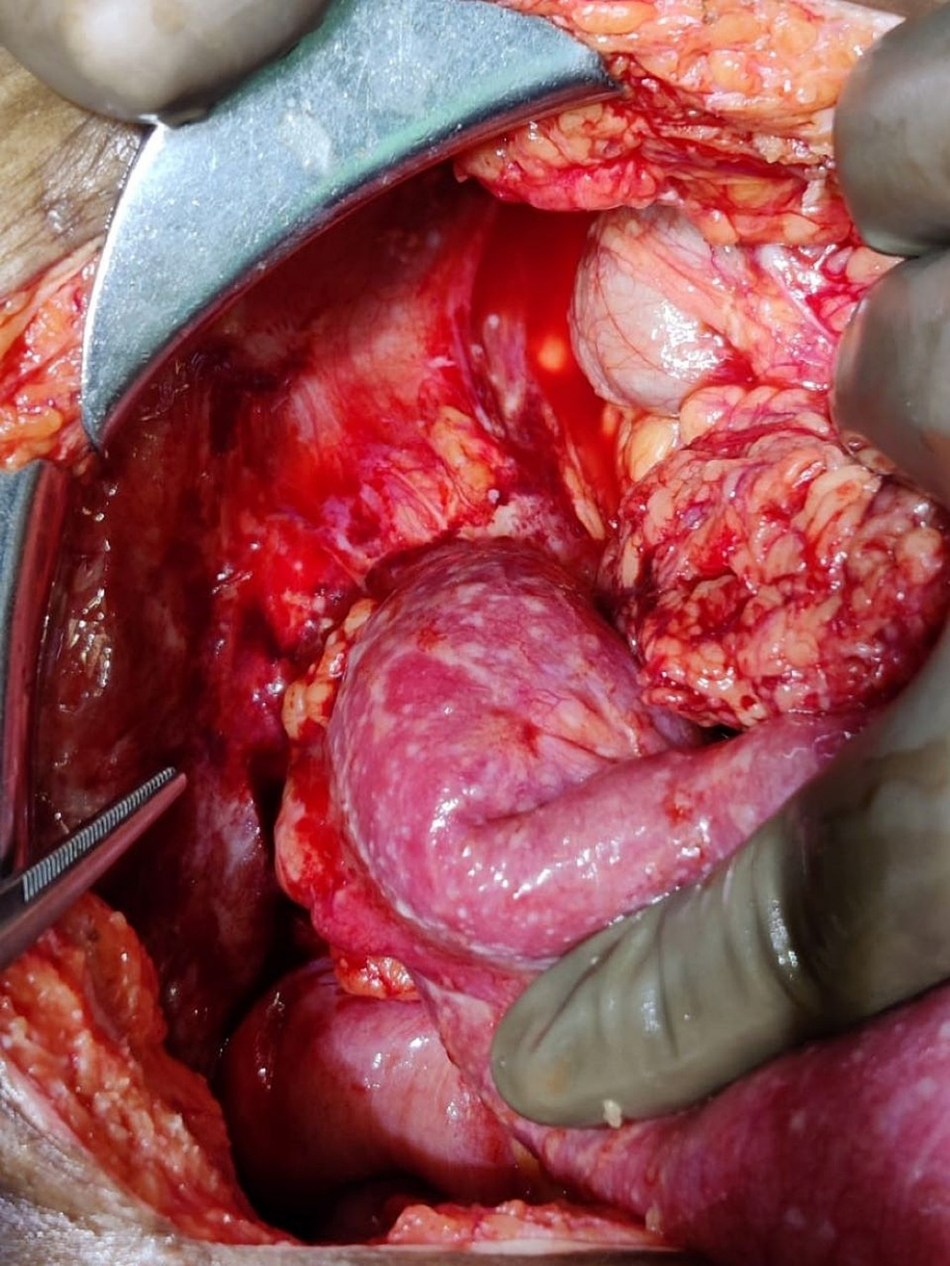
Sigmoid colon perforation with field contamination The instrument is pointing towards the post-ischemia gangrenous distal sigmoid colon segment, with surrounding fecal contamination.

**Figure 6:**
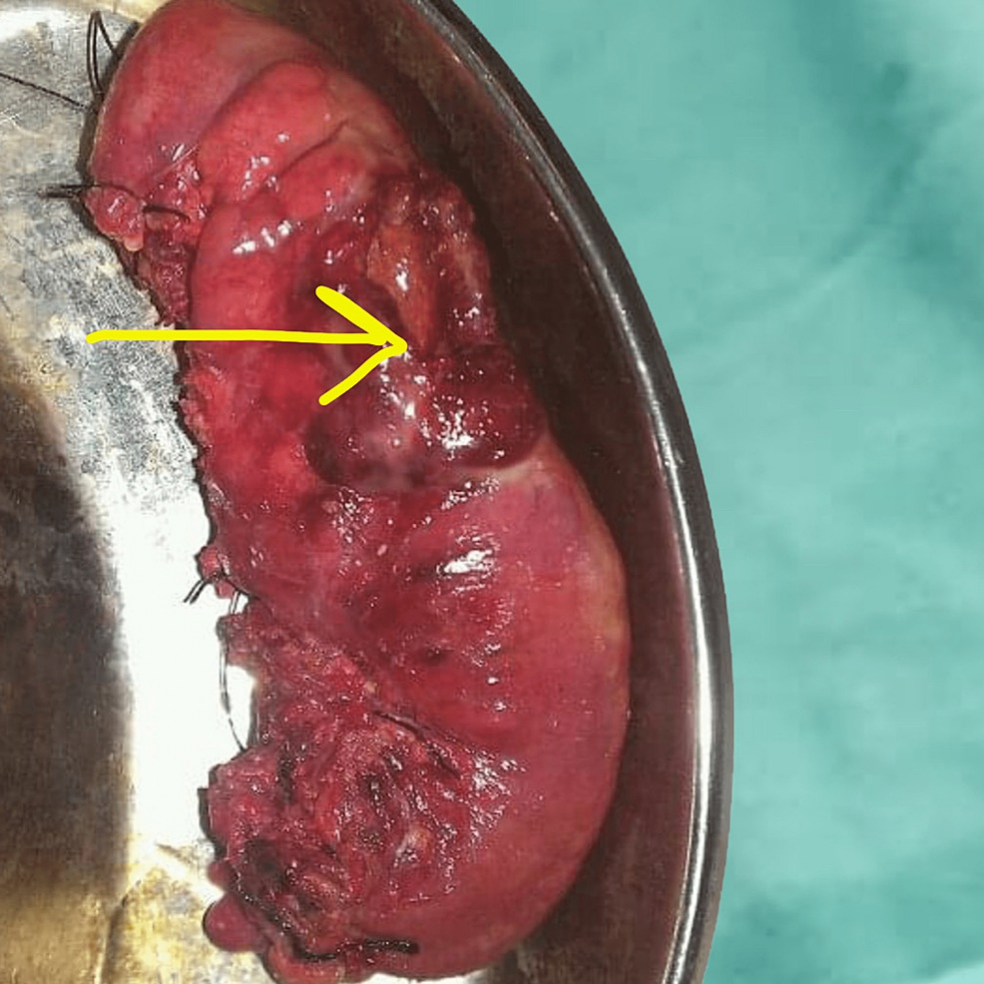
Resected distal sigmoid colon segment with perforation 2 x 1.5 cm perforation (arrow) over the posterior aspect of the distal sigmoid colon at antimesentric border.

Intraoperatively, 1500 ml sanguineous intraperitoneal fluid was aspirated and gross fecal contamination and mental clumping near the splenic flexure and sigmoid colon region were noted. The distal sigmoid segment was gangrenous (Figure 5) and a 2 x 1.5 cm perforation over the posterior aspect of the distal sigmoid colon at the antimesentric border was present (Figure 6). The rest of the intestine and other intraabdominal organs were normal. Based on the intraoperative assessment, due to gross intraabdominal contamination and hemodynamic instability, the decision to resect the perforated sigmoid colon segment with end colostomy (Hartman procedure) was made and the patient was shifted to the critical care unit for further resuscitation. Post-operative outcome was unremarkable. Histopathological examination revealed a gangrenous bowel segment with mesenteric ischemia and feculent coating over the serosa.

## Discussion

Isolated colon involvement is a relatively common occurrence in penetrating injuries to the abdomen, but a rare entity after blunt abdominal trauma, with the reported incidence being 0.1- 0.5% [1,2]. Road Traffic accidents are the most commonly reported cause of blunt colon trauma, with other common causes being a direct impact to the abdomen and a fall from height [1-3]. The transverse colon is the most frequently involved colon segment. Isolated sigmoid colon involvement is reported only in 34.8% of isolated colonic injuries [3]. In the majority of cases previously reported, an accompanying intra-abdominal solid organ injury was present [1-2], but this case series was devoid of any such association.

Proposed mechanisms for colon injuries post blunt abdominal trauma are (i) bowel crushing against vertebrae; (ii) rapid pressure accentuating involvement of specific narrow segments of the colon, and (iii) colonic/ mesentery shearing [1-5]. The resultant outcome is serosal tears, bowel perforations, bowel transection, mesenteric hematomas, serosal hematomas, ischemic injury, and gangrene of the colon [1-3,5]. The proposed reasons for isolated sigmoid perforation in blunt trauma abdomen are redundant sigmoid colon with long and wide mesentery and a loaded colon that gets perforated on sudden impact [6]. Diagnosis of isolated colonic injury following blunt abdominal trauma is often difficult due to associated multi-organ injuries and a paucity of signs requiring urgent laparotomy [2]. In such cases, the interval between presentation and operative intervention assumes importance in reducing morbidity and mortality in the postoperative period [2-3]. 

No clinical or radiological method has adequate accuracy in diagnosing blunt traumatic colon injuries [5]. Blood-stained gloves on digital rectal examination along with abdominal tenderness are poor indicators of colonic injury. Leukocytosis can be indicative of pathology [7]. Abdominal radiographs are often inconclusive; bowel edema and free fluid on ultrasonography can be suggestive of bowel injury. CECT has 64% sensitivity and 82% accuracy in predicting bowel injury [5], and though most indicative, has controversial diagnostic utility with only 20% accuracy for sigmoid colonic involvement [8]. However, CECT is significantly better than clinical examination and other investigational modalities (like DPL {diagnostic peritoneal lavage} and ultrasonography) which often lead to negative laparotomies in trauma cases [2,3].

Surgical treatment comprises primary closure of perforation, or resection of the perforated segment with/without anastomosis and/or colostomy formation [1-3]. Most reported literature is in favor of primary anastomosis whenever applicable [1-3], due to better suture line acceptance, prevention of stoma complications, and the need for a second operation. However, colostomy creation is required in cases of >50% wall circumference involvement, devascularization, full-thickness perforation, hemodynamic instability, or associated comorbidities [1-3], as colostomy creation was associated with decreased mortality in such subgroup [9]. In our case series, the decision was made by the operating surgeon based on intraoperative inflammation/contamination and perforated segment condition.

## Conclusions

Isolated sigmoid colon perforation post blunt trauma has a low reported incidence. Diagnostic dilemmas and treatment delays subsequently increase morbidity and mortality rates. Hence high index of suspicion is required to reach a prompt diagnosis. A specific definitive management algorithm is required to be formulated. Additional research is required to define the patient population likely to benefit from surgical maneuvers and explore the application of different surgical treatment options.
